# c-Rel is a critical mediator of NF-*κ*B-dependent TRAIL resistance of pancreatic cancer cells

**DOI:** 10.1038/cddis.2014.417

**Published:** 2014-10-09

**Authors:** C Geismann, F Grohmann, S Sebens, G Wirths, A Dreher, R Häsler, P Rosenstiel, C Hauser, J-H Egberts, A Trauzold, G Schneider, B Sipos, S Zeissig, S Schreiber, H Schäfer, A Arlt

**Affiliations:** 1 Laboratory of Molecular Gastroenterology and Hepatology, 1st Department of Internal Medicine I, University Hospital Schleswig-Holstein, Kiel, Germany; 2 Institute for Experimental Medicine, Kiel, Germany; 3 Institute of Clinical Molecular Biology, Kiel, Germany; 4 Department of Surgery, Kiel, Germany; 5 Division of Molecular Oncology, Institute for Experimental Cancer Research, Kiel, Germany; 6 Technische Universität München, Klinikum rechts der Isar, II. Medizinische Klinik, Munich, Germany; 7 Institute of Pathology, University Hospital Tübingen, Tübingen, Germany

## Abstract

Pancreatic ductal adenocarcinoma (PDAC) represents one of the deadliest malignancies with an overall life expectancy of 6 months despite current therapies. NF-*κ*B signalling has been shown to be critical for this profound cell-autonomous resistance against chemotherapeutic drugs and death receptor-induced apoptosis, but little is known about the role of the c-Rel subunit in solid cancer and PDAC apoptosis control. In the present study, by analysis of genome-wide patterns of c-Rel-dependent gene expression, we were able to establish c-Rel as a critical regulator of tumour necrosis factor-related apoptosis-inducing ligand (TRAIL)-induced apoptosis in PDAC. TRAIL-resistant cells exhibited a strong TRAIL-inducible NF-*κ*B activity, whereas TRAIL-sensitive cells displayed only a small increase in NF-*κ*B-binding activity. Transfection with siRNA against c-Rel sensitized the TRAIL-resistant cells in a manner comparable to siRNA targeting the p65/RelA subunit. Gel-shift analysis revealed that c-Rel is part of the TRAIL-inducible NF-*κ*B complex in PDAC. Array analysis identified NFATc2 as a c-Rel target gene among the 12 strongest TRAIL-inducible genes in apoptosis-resistant cells. In line, siRNA targeting c-Rel strongly reduced TRAIL-induced NFATc2 activity in TRAIL-resistant PDAC cells. Furthermore, siRNA targeting NFATc2 sensitized these PDAC cells against TRAIL-induced apoptosis. Finally, TRAIL-induced expression of COX-2 was diminished through siRNA targeting c-Rel or NFATc2 and pharmacologic inhibition of COX-2 with celecoxib or siRNA targeting COX-2, enhanced TRAIL apoptosis. In conclusion, we were able to delineate a novel c-Rel-, NFATc2- and COX-2-dependent antiapoptotic signalling pathway in PDAC with broad clinical implications for pharmaceutical intervention strategies.

Pancreatic ductal adenocarcinoma (PDAC) is one of the most malignant tumour diseases representing the fourth leading cause in the statistics of cancer-related death in western countries.^1^ With a five-year survival rate of under 5%, this tumour entity represents one of the deadliest tumours. As PDAC is generally diagnosed in a progressed tumour stage, only for 15% of the patients a curative intended resection is a therapeutic option. For the remaining majority of patients, current strategies of chemotherapy with or without radiation offer only limited gain in the life expectancy. At present, gemcitabine with erlotinib or FOLFIRINOX (folic acid, 5-fluorouracil, irinotecan, oxaliplatin) are the first-line therapeutic options in these settings. However, both offer just some marginal advantage over best supportive therapy and are highly dependent on the selection of the adequate treatment subgroup.^2,3^

Besides late diagnosis, a profound cell-intrinsic and -extrinsic resistance against chemotherapeutic drug and/or death receptor ligand-induced apoptosis contributes to this bad outcome. Several molecular alterations have been described over the past decade in this tumour entity. In addition to genetic alterations,^4^ a chronic inflammatory environment and the interaction of epithelial tumour cells with the surrounding tumour stroma are involved in PDAC development and apoptosis resistance.^5^ These alterations lead to a deregulated activity of several potentially oncogenic and inflammation related transcription factors, including NF-*κ*B, NFAT and Nrf2.^6,7^

A pivotal role in carcinogenesis and chemoresistance of a great number of tumours, including PDAC, has been shown for NF-*κ*B.^4,6,8, 9, 10^ This transcription factor is composed as a hetero- or homodimer of members of the so-called Rel family.^4,6,11^ Proteins of this family harbour a Rel homology domain, which mediates dimerization as well as the interaction with inhibitory components of the pathway. Five members of this family – RelA/p65, RelB, c-Rel, NF-*κ*B1 (p50/p105) and NF-*κ*B2 (p52/p100) – are known. In contrast to NF-*κ*B1 and NF-*κ*B2, RelA, RelB and c-Rel possess a C-terminal transactivation domain (TAD), which is essential for the transcriptional regulation.^4,6,11^ Two major pathways of NF-*κ*B activation have been reported so far. In the so-called classical or canonical pathway, inflammatory stimuli or death receptor ligands activate the I*κ*B kinase (IKK) complex. The activated IKK phosphorylates an inhibitor of *κ*B (I*κ*B), which in turn is polyubiquitinated and degraded by the 26 S proteasome. The pre-existing NF-*κ*B dimer, initially bound to I*κ*B, then translocates to the nucleus and modulates the expression of its target genes.^4,6,11^ Whilst tumour necrosis factor-related apoptosis-inducing ligand (TRAIL) and TNF-*α* mostly activate the canonical pathway, a subset of TNF receptor superfamily members activate the non-canonical or alternative pathway through NF-*κ*B-inducing kinase, which interacts with IKK*α* and in turns leads to the degradation of the p52 precursor protein p100. P100 functions as an inhibitor in the non-canonical pathway, and after its degradation, the most abundant dimer of this pathway, the p52/RelB dimer, can regulate its target genes. The non-canonical pathway is mainly involved in specific immunologic processes, but there is growing evidence that it might be involved in PDAC development and apoptosis resistance as well.^12, 13, 14, 15, 16, 17^

There is clear evidence that death receptor ligands and chronic inflammation, such as chronic pancreatitis, induce NF-*κ*B and thereby inhibit PDAC cell apoptosis. Moreover, PDAC cells gain growth advantage by auto- and paracrine loops involving inflammatory mediators such as IL-1*β* or TNF-*α*, and also through the treatment with the apoptotic stimulus itself.^18, 19, 20, 21, 22, 23, 24^ Especially, the death receptor ligands TNF-*α*, TRAIL and FASL induce NF-*κ*B,^20,25, 26, 27, 28, 29, 30^ and this inducible, but not constitutive NF-*κ*B activity^31^ limits apoptosis induced by death receptor signalling in PDAC cells.^4,6^ In contrast to the well-established role of RelA and the increasing number of reports on RelB in PDAC and in solid cancer biology in general, little is known about the other transactivation domain (TAD) harbouring Rel subunit, c-Rel. So far, just a few studies have described a role of c-Rel in solid cancer including head and neck or mammary cancer.^32, 33, 34^

In the present study, we explored a possible role of c-Rel in NF-*κ*B-mediated apoptosis resistance of PDAC.^35, 36, 37^ We describe a novel pathway involved in dampening death receptor-induced apoptosis and show how such a resistance mechanism can be addressed pharmacologically.

## Results

### TRAIL sensitivity correlates with basal and inducible NF-*κ*B activity in PDAC cell lines

**Figure 1:**
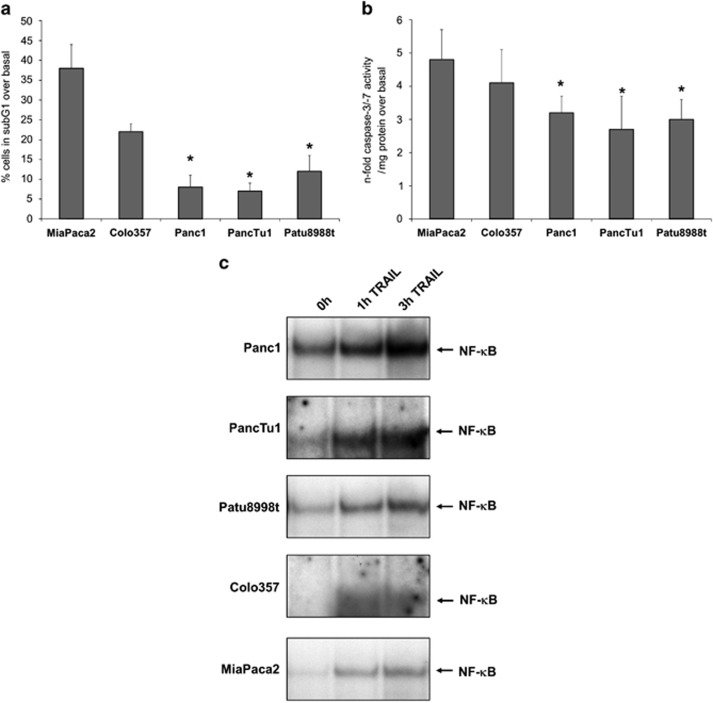
TRAIL sensitivity correlates with NF-*κ*B activity in PDAC cell lines. (**a** and **b**) Cells were treated with 10 ng/ml TRAIL for 24  and 5 h, respectively. Apoptosis was determined by analysing sub-G1 content (**a**) or caspase-3/-7 activity (**b**). Data are presented as % of sub-G1 content over basal (untreated cells) or expressed as *n*-fold caspase-3/-7 activity normalized to the cellular protein content and represent the mean value±S.D. from six independent experiments. *P-values <0.05. (**c**) Cells were left untreated or were treated with 10 ng/ml TRAIL for the indicated time periods and submitted to EMSA for measurement of NF-*κ*B-binding activity. All samples was loaded on the same gel to compare the binding activity. Only for the presentation each cell line is presented as a single row but shows the same amount of total nuclear protein and the same exposition time. A representative of four independent experiments is shown

To characterize the sensitivity to TRAIL, several well-established PDAC cell lines (Panc1, PancTu1, Patu8988t, Colo357 and MiaPaca2) were subjected to TRAIL treatment and subsequent apoptosis analyses. MiaPaca2 cells were highly sensitive to TRAIL. After 24 h of treatment, 38% of MiaPaca2 cells were detected in the sub-G1 phase representing apoptotic cells (Figure 1a), and caspase-3/-7 activity was increased by 4.8-fold over basal activity after 5 h TRAIL treatment (Figure 1b). The Colo357 cell line showed a moderate sensitivity for this treatment with 22% of the cells in the sub-G1 phase (Figure 1a) and caspase-3/-7 activity was increased by 4.1-fold over basal activity after 5 h TRAIL treatment (Figure 1b). In contrast, Panc1, PancTu1 and Patu8988t were resistant to TRAIL treatment. Only 8% of Panc1, 7% of PancTu1 and 12% of Patu8988t cells showed signs of apoptosis detectable in the sub-G1 fraction (Figure 1a), and the three cell lines exhibited a smaller increase (Panc1=3.2-fold, PancTu1=2.7-fold and Patu8988t=3.0-fold) of caspase-3/7 activity (Figure 1b).

EMSA revealed a high basal NF-*κ*B activity in Panc1 cells and a lower basal activity in PancTu1 and Patu8988t cells (Figure 1c). Compared with these cell lines, Colo357 and MiaPaca2 cells had nearly no detectable basal NF-*κ*B-binding activity. TRAIL induced a transient induction of NF-*κ*B in all cell lines tested with a maximum after 3 h. The overall binding activity of NF-*κ*B measured by EMSA remained higher in the Panc1, PancTu1 and Patu8988t cells at every time point compared with the MiaPaca2 cell line (Figure 1c). In line with the apoptotic response, the Colo357 cell showed slightly higher NF-*κ*B activity than the MiaPaca2 cell line but lower activities than the resistant cell lines (Figure 1c).

### All Rel subunits are involved in TRAIL-induced NF-*κ*B and apoptosis response

To investigate the role of RelA, RelB and c-Rel in PDAC, we choose two resistant cell lines (Panc1 and Patu8988t) and the sensitive MiaPaca2 cell line and established Rel subunit-specific siRNA-mediated knockdown with two sets of siRNAs.

**Figure 2:**
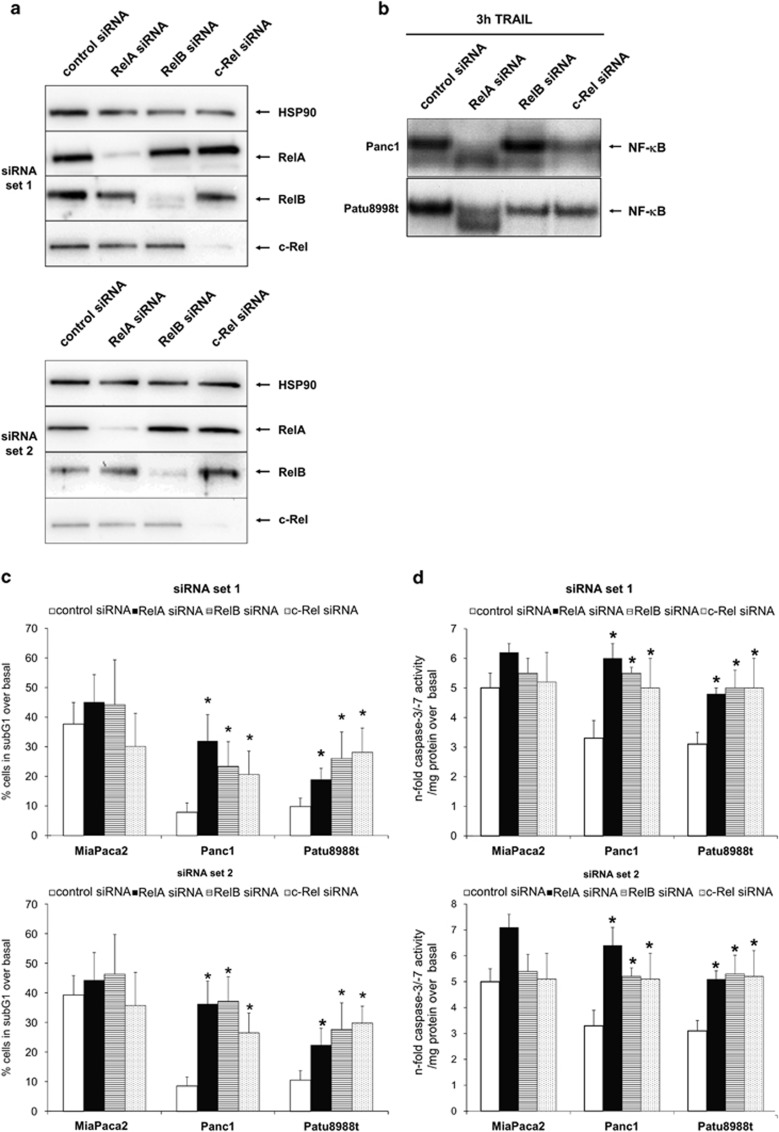
All Rel subunits are involved in TRAIL-induced NF-*κ*B and apoptosis. (**a**) Whole-cell extracts of Panc1 cells transfected with the indicated siRNA for 48 h were submitted to western blotting for the indicated Rel subunit and Hsp90 as the loading control. A representative of four independent experiments with two different sets of siRNA is shown. (**b**) Cells were transfected with the indicated siRNA (set1) for 48 h, treated with 10 ng/ml TRAIL for 3 h and nuclear extracts were submitted to EMSA for measurement of NF-*κ*B-binding activity. A representative of four independent experiments is shown. (**c** and **d**) Cells were transfected with with two different sets of siRNA (as indicated) for 48 h and then treated with 10 ng/ml TRAIL for 24 and 5 h, respectively. Apoptosis was determined by analysing sub-G1 content (**c**) or caspase-3/-7 activity (**d**). Data are presented as % of sub-G1 content over basal (untreated cells) or expressed as *n*-fold caspase-3/-7 activity normalized to the cellular protein content and represent the mean value±S.D. from six independent experiments. *P-values <0.05

As shown by western blot analysis (Figure 2a), the chosen siRNA mediated a specific knockdown of the targeted Rel subunit without effects on one of the other subunits.

Next, EMSAs were conducted to determine the effects of the specific knockdown on NF-*κ*B–DNA complex formation (Figure 2b) in the resistant cell lines. In Panc1 cells, siRNA directed against RelA or c-Rel strongly reduced NF-*κ*B-binding activity after 3 h TRAIL treatment, whereas siRNAs targeting RelB had no detectable effect. In Patu8988t cells, downregulation of either of the three Rel subunits reduced the observed NF-*κ*B-binding activity. In both cell lines, siRNA against RelA led to an enhancement of a lower band that is known to represent p50 homodimers.^9,22,27^

We next assed apoptosis after knockdown of Rel subunits. In TRAIL-sensitive MiaPaca2 cells, knockdown of either of the Rel subunits had only marginal effect on TRAIL responsiveness (Figures 2c and d). In contrast, the resistant cell lines were sensitized to TRAIL if either one of the Rel subunits was knocked down. As shown by sub-G1 analysis (Figure 2c) and caspase-3/-7 activity (Figure 2d), apoptotic cell death after TRAIL treatment was increased by 2–3-fold through the knockdown of RelA, RelB or c-Rel compared with control siRNA-transfected cells.

### NFATc2 is a c-Rel target gene in TRAIL-resistant Panc1 cells

An unbiased, genome-wide transcriptome analysis was performed to identify antiapoptotic c-Rel target genes involved in TRAIL resistance. As inducible NF-*κ*B activity is thought to be more relevant for TRAIL resistance,^4,6^ we focused on genes differentially induced by TRAIL in c-Rel-proficient and -deficient cells. Samples from the two cell lines with the highest and lowest TRAIL-inducible NF-*κ*B activity (Figure 1c) and TRAIL-inducible apoptotic response (Figures 1a and b) left untreated or stimulated 5 h with TRAIL were included in this analysis. This time point was chosen because of the maximal NF-*κ*B DNA-binding activity seen 3 h after TRAIL stimulation (Figure 1c) and the maximum of mRNA induction seen 5 h after TRAIL stimulation, as verified by two classical NF-*κ*B target genes IL-8 and I*κ*B*α* (data not shown).

**Figure 3:**
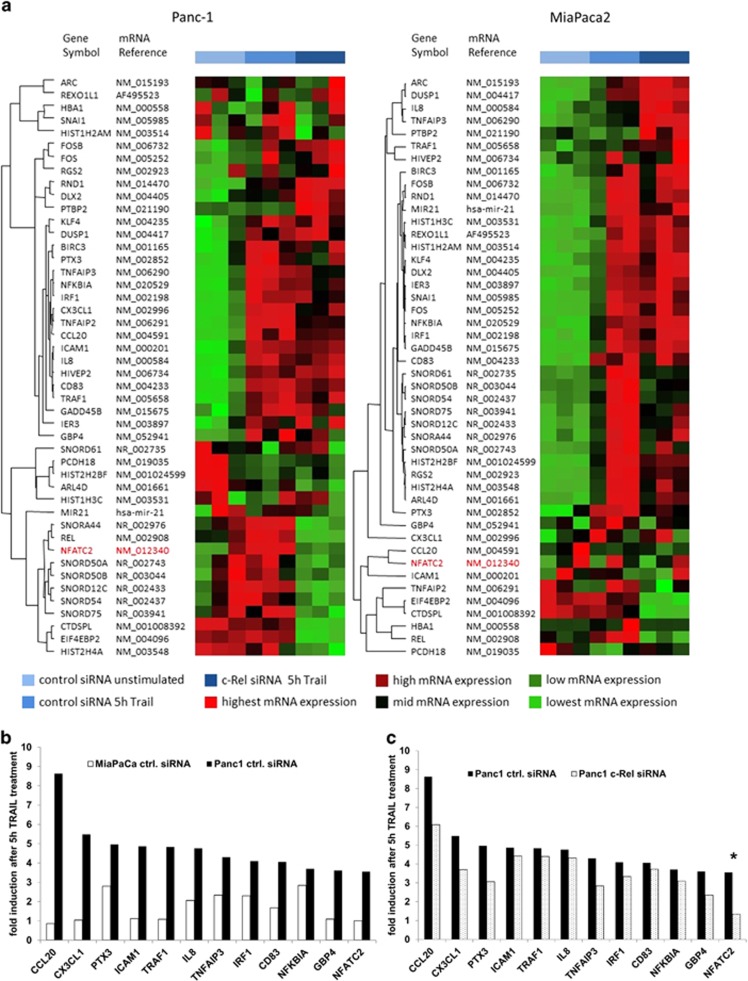
NFATc2 is a c-Rel target gene in TRAIL-resistant Panc1 cells. (**a**) Hierarchical clustering of top 50 differentially expressed genes in Panc1 cells and MiaPaca2 cells. Transcripts were selected for being differentially expressed in either of the cell lines and associated with characterized genes. Transcripts are displayed in rows and samples are listed in columns, whereas the row dendogram represents the similarity between the individual transcripts. Transcription levels and experimental groups are colour coded. Hierarchical clustering was performed using the unweight pair group method with arithmetic mean as a cluster algorithm, and similarities between transcripts are based on their Spearman rank correlation. For better readability, all transcripts were *z*-score normalized before cluster analysis. (**b**) Transcript levels were normalized to unstimulated cells and the 12 genes with the highest fold induction in the resistant Panc1 cells transfected with the control siRNA (black columns) are presented in comparison with the sensitive MiaPaca2 cell line transfected with the control siRNA (white columns). Data represent the mean of three independent experiments. (**c**) Transcript levels were normalized to unstimulated cells and the effect of the transfection of resistant Panc1 cells with the c-Rel siRNA in comparison with the resistant Panc1 cells transfected with the control siRNA are presented. Data represent the mean of three rindependent experiments. *P-values <0.05 for the effect of the c-Rel siRNA on TRAIL-mediated gene induction

Cluster analysis of the 50 most strongly TRAIL-induced genes identified substantial differences and heatmap analysis clearly indicated a differential TRAIL-inducible genetic network in Panc1 and MiaPaca2 cells (Figure 3a). The group of transcripts that exhibits the strongest regulation upon TRAIL stimulation expectedly contained IL-8 and I*κ*B*α*, which were 4–5-fold induced in Panc1 and only 2–3-fold induced in MiaPaca2 cells, confirming their divergent NF-*κ*B responsiveness to TRAIL treatment (Figure 3b). By comparing the induction of these genes in control and c-Rel siRNA-transfected Panc1 cells, the transcription factor NFATc2 was identified as the one upregulated gene being mostly affected by c-Rel knockdown in Panc1 cells (Figure 3c). A 3.6-fold induction in control siRNA-transfected Panc1 cells was observed that was almost extinguished (1.3-fold induction) in c-Rel siRNA-transfected Panc1 cells (Figure 3c). Since having been reported to be involved in several aspects of PDAC carcinogenesis^35,37,38^ and because none of the other top 50 TRAIL-inducible genes (Figure 3b) showed a comparable c-Rel dependency, we focused our interest on NFATc2.

### TRAIL-induced NFATc2 expression and NFATc2 DNA binding are c-Rel dependent

**Figure 4:**
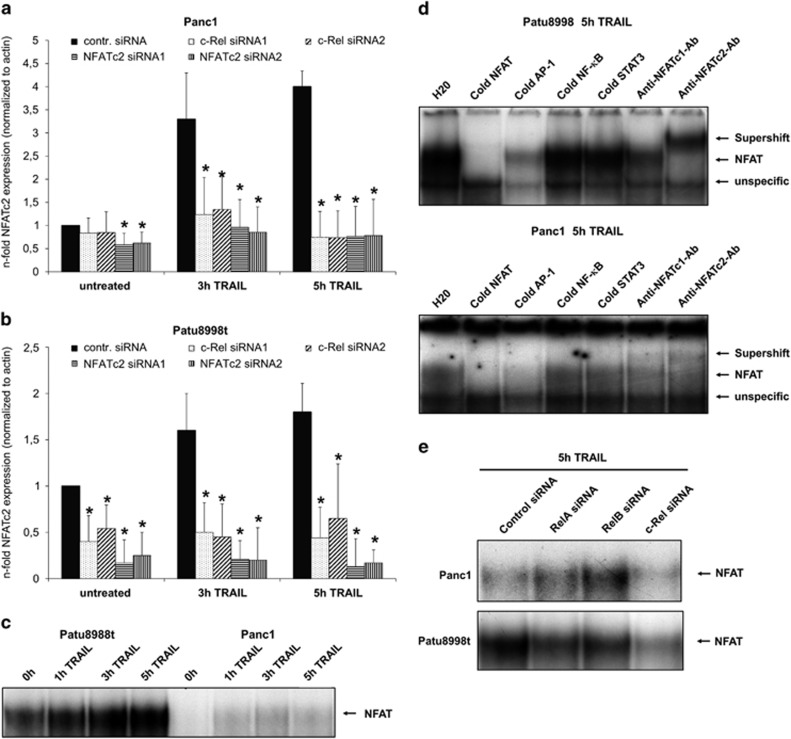
TRAIL-induced NFATc2 expression and DNA binding are c-Rel dependent. (**a** and **b**) Panc1 (**a**) and Patu8998t (**b**) cells were transfected with the indicated siRNA for 48 h. Then cells were left untreated or treated with 10 ng/ml TRAIL for 3 or 5 h. Total RNA was submitted to reverse transcription and real-time PCR detecting NFATc2. For normalization, *beta*-actin was analysed as control. Data are expressed as normalized mRNA level and represent the mean values±S.D. from four independent experiments performed in duplicates. *P-values <0.05. (**c**) Patu8998t (left part) and Panc1 (right part) cells were left untreated or were treated with 10 ng/ml TRAIL for the indicated time periods and submitted to EMSA for measurement of NFAT-binding activity. A representative of four independent experiments is shown. (**d**) Nuclear extracts from Panc1 (upper part) or Patu8998t cells (lower part) treated for 5 h with 10 ng/ml TRAIL were submitted to EMSA with the indicated antibodies and was performed using the indicated oligonucleotides. A representative of four independent experiments is shown. (**e**) Panc1 (upper part) and Patu8998t (lower part) cells were transfected with the indicated siRNA (set1) for 48 h, and then treated with 10 ng/ml TRAIL for 5 h and submitted to EMSA for measurement of NFAT-binding activity. A representative of four independent experiments is shown

To confirm the observed effects of c-Rel on TRAIL-inducible NFATc2 expression (Figures 3a–c) and a possible role of this gene in TRAIL resistance, we next analysed the mRNA expression of NFATc2 in control, c-Rel and NFATc2 siRNA-transfected Panc1 and Patu8988t cells (Figures 4a and b). In both cell lines, TRAIL- induced NFATc2 mRNA expression – exhibiting a maximum after 5 h TRAIL treatment – was equally blocked by NFATc2 or c-Rel knockdown, confirming the role of c-Rel in the regulation of NFATc2 expression. Interestingly, c-Rel siRNA transfection also significantly reduced the basal NFATc2 mRNA expression in Patu8988t cells (Figure 4b). As this cell line is known to exert high basal NFATc2 activity, in contrast to Panc1 cells,^35,37,38^ we next analysed the basal and the TRAIL-inducible NFAT activity in both cell lines, using the established NFAT/AP-1 binding site of the COX-2 promoter, by EMSA (Figures 4c–e). As shown in Figure 4c, Patu8998t but not Panc1 cells revealed high basal NFAT activity, whereas TRAIL induced an increase in NFAT DNA binding in both cell lines. Supershifts (Figure 4d) confirmed that the DNA-binding complex in both cell lines consisted of NFATc2 (visible supershift) and to a lower content of NFATc1 (reduction of the shifted band). As expected, competition with a cold NFAT or AP-1 consensus probe completely reduced the binding activity. In contrast, an NF-*κ*B consensus sequence or an unrelated oligonucleotide harbouring a STAT3 binding site had no effect (Figure 4d).

To confirm the effects of c-Rel knockdown on protein level, EMSA with control or c-Rel siRNA-transfected cells were conducted, and unspecific interference of NF-*κ*B modulation was excluded by siRNAs targeting RelA or RelB. Indeed, only siRNA directed against c-Rel was capable of strongly reducing the NFAT-binding activity in Panc1 and Patu8988t cells (Figure 4e).

### Characterization of a functional c-Rel binding site in the NFATc2 promoter

**Figure 5:**
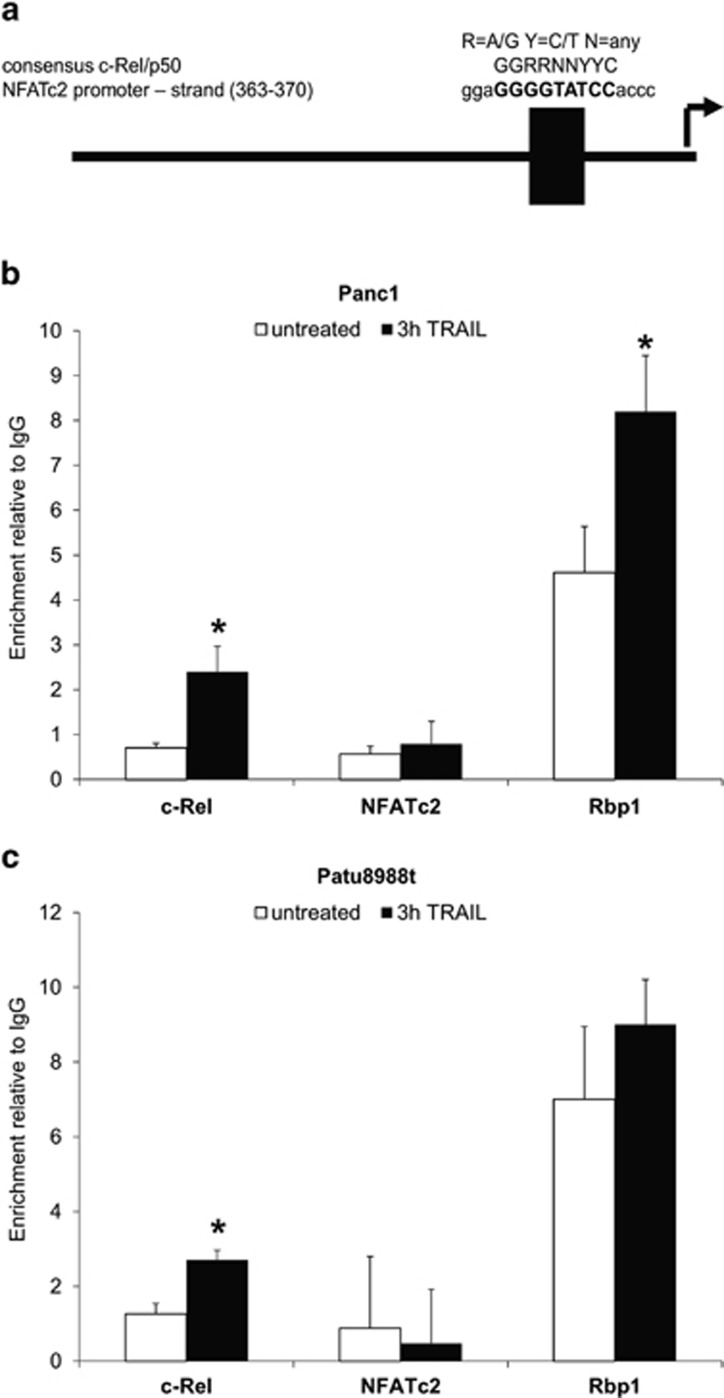
Characterization of a functional c-Rel binding site in the NFATc2 promoter. (**a**) Schematic representation of the putative c-Rel binding site (MatInspector query). For comparison, the c-Rel/p50 consensus site GGRRNNYYC (where R is for A/G, Y for C/T and N for any base) is shown. The putative binding motive in the NFATc2 promoter is highlighted in bold. (b and **c**) Panc1 (**b**) or Patu8998t cells (**c**) were left untreated (withe columns) or treated with 10 ng/ml TRAIL for 3 h (black columns) and qChIP were conducted with IgG, anti-c-Rel, anti-NFATc2 and anti-Rbp1 antibodies. Data are expressed as enrichment relative to IgG levels and represent the mean values±S.D. from six independent experiments performed in duplicates. *P-values <0.05

To identify the regulatory component for c-Rel-mediated NFATc2 expression, a database search using MatInspector (MatInspector Genomatix Software GmbH, Munich, Germany) for putative c-Rel binding motifs was performed. Hereby, we were able to identify one possible regulatory element at position −360 to −370 of the minus strand in the promoter region (Figure 5a), which showed high similarity to the published c-Rel/p50 consensus site.^39^

Quantitative chromatin immunoprecipitation (qChIP) assay revealed an anti-c-Rel-dependent enrichment of this site 3 h after TRAIL stimulation in Panc1 (2.4-fold; Figure 5b) and in Patu8988t cells (2.7-fold; Figure 5c). Moreover, recruitment of DNA polymerase II to this promoter region also increased in both cell lines 3 h after TRAIL stimulation, demonstrating transcriptional activation. In contrast, no enrichment of this site by an unspecific antibody (anti-NFATc2) (Figures 5b and c) was observed, demonstrating specificity. Taken together, qChIP assays verified NFATc2 as a novel c-Rel target gene.

### NFATc2 and COX-2 are downstream mediators of c-Rel in TRAIL resistance

**Figure 6:**
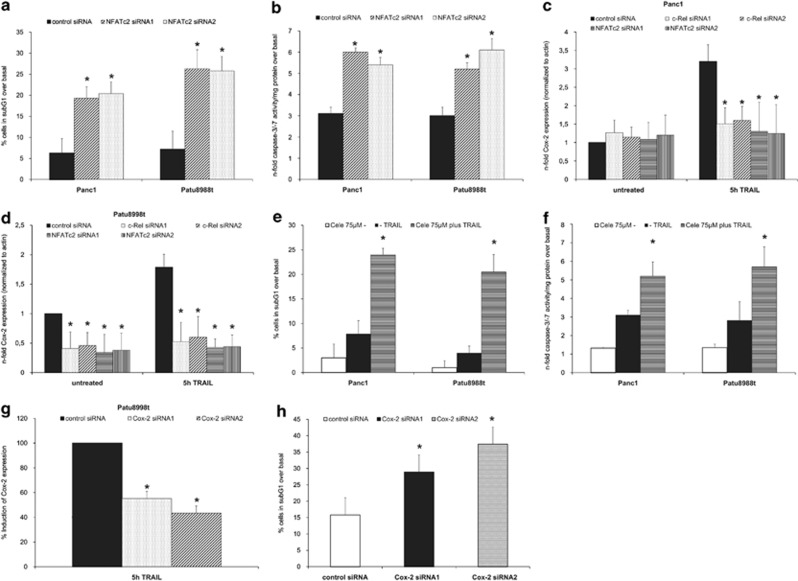
NFATc2 and COX-2 are downstream mediators of c-Rel in TRAIL resistance. (**a** and **b**) Cells were transfected with control or NFATc2 siRNA for 48 h and treated with 10 ng/ml TRAIL for 24 and 5 h, respectively. Apoptosis was determined by analysing sub-G1 content (**a**) or caspase-3/-7 activity (**b**). Data are presented as % of sub-G1 content over basal activity (untreated cells) or expressed as *n*-fold caspase-3/-7-activity normalized to the cellular protein content and represent the mean value±S.D. from six independent experiments. (**c** and **d**) Panc1 (**c**) and Patu8998t (**d**) cells were transfected with the indicated siRNA for 48 h. Then, cells were left untreated or treated with 10 ng/ml TRAIL for 5 h. Total RNA was submitted to reverse transcription and real-time PCR detecting COX-2. For normalization, *beta*-actin was analysed as control. Data are expressed as normalized mRNA level and represent the mean values±S.D. from four independent experiments performed in duplicates. *P-values <0.05. (**e** and **f**) Cells were pretreated with 75 *μ*M celecoxib (Cele) for 1/2 h and then treated with 10 ng/ml TRAIL for 24 and 5 h, respectively. Apoptosis was determined by analysing sub-G1 content (**e**) or caspase-3/-7 activity (**f**). Data are presented as % of sub-G1 content over basal activity (untreated cells) or expressed as *n*-fold caspase-3/-7 activity normalized to the cellular protein content and represent the mean value±S.D. from six independent experiments. *P-values <0.05. (**g**) Patu8998t cells were transfected with control or COX-2 siRNA for 48 h. Then, cells were left untreated or treated with 10 ng/ml TRAIL for 5 h. Total RNA was submitted to reverse transcription and real-time PCR detecting COX-2. For normalization, *beta*-actin was analysed as control. Data are expressed % induction of COX-2 mRNA with control siRNA-transfected cells set as 100% and represent the mean values±S.D. from four independent experiments performed in duplicates. *P-values <0.05. (**h**) Cells were transfected with control or COX-2 siRNA for 48 h and treated with 10 ng/ml TRAIL for 24 and 5 h, respectively. Apoptosis was determined by analysing sub-G1 content. Data are presented as % of sub-G1 content over basal activity (untreated cells) and represent the mean value±S.D. from six independent experiments

To investigate whether c-Rel induced NFATc2 expression is involved in TRAIL resistance, we analysed the effect of siRNA-mediated knockdown of NFATc2 in TRAIL-induced PDAC apoptosis. Sub-G1 analysis (Figure 6a) and caspase-3/-7 assays (Figure 6b) showed that siRNA against NFATc2 strongly enhanced the apoptotic response to TRAIL in both cell lines.

Based on the observation that NFATc2 binds to the NFAT site from the COX-2 promoter in the analysed PDAC cell lines (Figure 4) and owing to the fact that COX-2 is involved in several aspects of PDAC carcinogenesis,^40^ we next analysed the role of c-Rel and NFATc2 in COX-2 expression. Consistent with a linear c-Rel–NFATc2–COX-2 pathway, siRNA-mediated knockdown of c-Rel or NFATc2 strongly reduced the TRAIL-induced COX-2 expression in Panc1 and Patu8988t cells (Figures 6c and d). In Patu8988t, which exhibit high basal NFATc2 activity, siRNA directed against either one of the two transcription factors already reduced basal COX-2 mRNA levels (Figure 6d). In addition, in c-Rel or NFATc2 siRNA-transfected Patu8988t cells, TRAIL-induced COX-2 expression was significantly lower than in control siRNA-transfected Patu8988t cells (Figure 6d).

By using the clinically established COX-2 inhibitor celecoxib, we substantiated the functional role of COX-2 as a downstream mediator of c-Rel and NFATc2-mediated TRAIL resistance. Cells treated with celecoxib in combination with TRAIL (Figure 6e) exhibited significantly greater cell counts in the sub-G1 fraction (Panc1: 20–25% Patu8988t: 20%) compared with cells treated with TRAIL alone (Panc1: 8% Patu8988t: 4%). Caspase-3/-7 assays (Figure 6f) verified this greater number of apoptotic cells in the combination group (Panc1: 5-fold; Patu8988t: 5–6-fold) compared with the TRAIL group (Panc1: 3-fold; Patu8988t: 2–3-fold), demonstrating that the c-Rel-NFATc2 mechanism of TRAIL resistance can be addressed pharmacologically. To confirm that the effects of celecoxib on TRAIL-induced apoptosis were indeed mediated through COX-2 inhibition, we inhibited COX-2 expression through siRNA. As all tested siRNA targeting COX-2 had limited effeteness in reducing basal COX-2 expression data are expressed as relative induction of COX-2 after 5 h TRAIL treatment (Figure 6g). In line with the observation for celecoxib, siRNA-mediated knockdown of COX-2 expression significantly sensitized the resistant Patu8988t cells for TRAIL treatment (Figure 6h).

## Discussion

For the majority of PDAC patients, only palliative therapeutic strategies with limited effects on the life expectancy exist.^2,4^ Although the role of RelA in different aspects of PDAC is well established^4,6,14,41^ and recent reports indicate a role of RelB in PDAC development, growth and apoptotic response,^14, 15, 16^ only limited reports on such a role of c-Rel in other solid cancers^42, 43, 44^ and none in PDAC exist. Thus, the impact of c-Rel-directed genetic networks, especially on apoptosis resistance in PDAC, is not understood.

In our cell-based models of PDAC, we were able to show for the first time that c-Rel is a central mediator of an antiapoptotic signalling pathway protecting from TRAIL-induced apoptosis. By siRNA-mediated downregulation of c-Rel expression, we were able to show that c-Rel is part of the NF-*κ*B complex induced by TRAIL and conferring apoptosis resistance to an extent similar to other NF-*κ*B family members. As initially shown by array analysis and later confirmed by qPCR and EMSA, c-Rel induces the expression of the transcription factor NFATc2 in TRAIL-resistant PDAC cell lines. SiRNA-mediated downregulation of either c-Rel or NFATc2 significantly sensitized PDAC cell lines to TRAIL-induced apoptosis and abolished the induction of COX-2 expression by TRAIL in these cell lines. In line with this, pharmacologic inhibition of COX-2 by celecoxib greatly sensitized the PDAC cell lines in an extent similar to c-Rel or NFATc2 inhibition.

Unlike a recent study showing comparable effects of c-Rel on the apoptotic response in head and neck cancer,^32,33^ we did not observe any changes in proapoptotic genes such as *PUMA* or *p21* in the array analysis. Furthermore, we were not able to confirm any change in the expression of the death receptors for TRAIL in the PDAC lines,^45^ neither by array nor by FACS analysis (data not shown). In contrast to reports indicating a proapoptotic function of c-Rel,^46,47^ we clearly established an antiapoptotic effect of c-Rel in TRAIL-resistant PDAC cell lines.

To elucidate the involved target genes of the observed antiapoptotic c-Rel pathway, we analysed the group of transcripts that exhibited the strongest differential regulation upon 5 h TRAIL stimulation in Panc1 and MiaPaca2 cells. Hereby, we were able to show that the transcription factor NFATc2 was the gene most affected by siRNA-mediated knockdown of c-Rel in Panc1 cells. This transcription factor has been reported to be involved in several aspects of PDAC carcinogenesis.^35,37,38^ A recent study reported a high expression of NFATc2 in PDAC and a possible role in resistance against chemotherapeutic drugs.^48^ The observed high expression of NFATc2 in Patu8998t cells has also been reported by other groups.^35,37,38^ In concordance with the published data, Panc1 had only low basal NFATc2 levels, but an induction of NFATc2 was also observed in this cell line. It can be therefore speculated that the differences in the basal NFATc2 expression explain the effects of the c-Rel or NFATc2 siRNA on the basal COX-2 expression in Patu8998t cells.

In line with several other reports on NFAT family members in solid cancer, the NFATc2 protein is mainly localized in the nucleus in the PDAC cell lines.^35,37,48^ Similar to the role of c-Rel in apoptosis regulation, there are controversial reports on the role of NFATc2 in apoptosis and growth control, as well. Some studies show a proapoptotic function of NFAT, which is in part mediated by an RAS-dependent pathway.^49^ Other reports clearly describe an antiapoptotic proliferative effect of NFATc2 activity.^35,37,48^ By siRNA-mediated inhibition of NFATc2 signalling and by using an oligonucleotide harbouring the AP-1/NFAT site from the COX-2 promoter, we were able to show that NFATc2 is involved in the observed upregulation of COX-2. Such an NFAT-mediated upregulation of COX-2 through the proximal AP-1/NFAT site has been reported recently^50, 51, 52^ for other solid tumours. Interestingly, a functional interaction between the NF-*κ*B and the NFAT pathways in nickel-induced COX-2 expression in lung cancer has been described,^53^ also showing a not understood dependency of NF-*κ*B activity for NFAT-mediated COX-2 induction. Finally, the fact that celecoxib sensitized the Panc1 and Patu8998t cells for TRAIL-induced apoptosis showed the functional relevance of this c-Rel/NFATc2/COX-2 signalling pathway. As cell-intrinsic therapy resistance is one of the major obstacles for the existing novel therapies and for contribution to the failure of many clinical trials with PDAC patients in the past,^54^ investigations of such resistance programs are a suitable approach to develop novel therapeutic strategies. Here, we provide evidence for the first time that a c-Rel/NFATc2/COX-2 pathway confers resistance of PDAC toward TRAIL agonists. Considering current clinical trials using TRAIL agonistic ligands^55^ and the reported resistance problems in PDAC patients,^4,56,57^ the described pharmacologic approach using the clinically established COX-2 inhibitor celecoxib could markedly increase the efficacy of these approaches in PDAC. There is firm evidence that NSAID-mediated inhibition of COX-2 activity can sensitize PDAC cells for apoptosis-inducing agents *in vivo*.^58, 59, 60^ Currently, pentoxifylline could be used to pharmacologically target c-Rel nuclear translocation^61^ and also to inhibit NFAT,^62^ but without reported direct effects on COX-2. In line with this, preliminary *in vitro* results (data not shown) demonstrated an apoptosis sensitization by pentoxifylline.

In summary, we identified a c-Rel/NFATc2/COX-2 pathway eliciting apoptosis resistance against TRAIL treatment in PDAC that may serve as pharmacologic target.

## Materials and Methods

### Materials

Cell culture medium was purchased from Biochrom (Berlin, Germany), foetal calf serum (FCS) from Biochrom, horse serum (HS) from Life Technologies (Darmstadt, Germany), *Killer*-TRAIL was from Enzo Life Science/Alexis (Lörrach, Germany) and celecoxib from LKT Laboratories (St. Paul, MN, USA).

### Cell culture

The human PDAC cell line Panc1 (ATCC (Manassas, VA, USA)/LSC) was cultured in RPMI-1640 medium containing 10% FCS, 1% L-glutamine and 1% sodium pyruvate (all from Biochrom), Patu8988t (DSMZ, Braunschweig, Germany) cells in DMEM (high glucose) containing 10% FCS, 1% L-glutamine and 5% HS. MiaPaca2 cells (ATCC/LSC) were cultured in DMEM (high glucose) supplemented with 10% FCS, 2.5% HS and 1% L-glutamine. Handling of PancTu1^63^ and Colo357 cells^56^ were carried out as described recently. Cells were incubated at 37 °C with 5% CO_2_ at 85% humidity.

### Western blotting

Preparation of nuclear extracts or total cell lysates was carried out as described before.^64^ After electrophoresis and wet electroblotting onto PVDF membranes, the following primary antibodies were used for immunodetection at a 1000-fold dilution in 5% (w/v) non-fat milk powder, 0.05% Tween-20 in TBS (Tris-buffered saline; 50 mM Tris-HCl, pH 7.6, and 150 mM NaCl): RelA/p65 (sc-372G), RelB (sc-226), c-Rel (sc-671), NFATc2 (sc-7296) and Hsp90 (all from Santa Cruz Biotechnology, Heidelberg, Germany). After incubation overnight at 4 °C, blots were exposed to the appropriate horse radish peroxidase-conjugated secondary antibody (Santa Cruz Biotechnology) diluted (1 : 1000) in blocking buffer and developed using the Dura Detection Kit (Perbio Sciences, Bonn, Germany). Data acquisition was carried out with the Chemidoc-XRS gel documentation system (Bio-Rad, Munich, Germany) using the Quantity One software (Bio-Rad). Hsp90 served as the loading control.

### Gel-shift assay

Gel-shift assays on nuclear extracts were performed as described previously.^64^ For the detection of NF-*κ*B, a consensus *γ*-^32^P-labelled NF-*κ*B oligonucleotide (Promega, Mannheim, Germany) was used, for detection of NFAT a *γ*-^32^P-labelled oligonucleotide harbouring the proximal binding sequence of the cox-2 promoter or the consensus of the IL-2 promoter^52^ purchased from Biometra (Göttingen, Germany) were used. For control experiments, cold competition with AP-1 consensus oligonucleotide (Promega) or STAT3 (5′-GATCCTTCTGGGAATTCCTAGATC-3′) (Biometra) oligonucleotides, as well as supershift experiments with 4 *μ*g of NFATc2 (sc7295X) and NFATc1 (sc7294X) antibodies (both Santa Cruz Biotechnology) were performed.

### RNA preparation and real-time PCR

Isolation of total RNA and reverse transcription into single-stranded cDNA was carried out as described.^64^ cDNA was subjected to real-time PCR (iCycler; Bio-Rad) using the SYBR-Green assay with gene-specific primers at a final concentration of 0.2 *μ*M. Primers used for the determination of mRNA expression levels were: NFATc2-F (5′-CACGGGGCAGAACTTTACAT-3′), NFATc2-R (5′-CCCAAATTTGCTGTCCATCT-3′); *β*-actin-F (5′-CTCTTCCAGCCTTCCTTCCT-3′); *β*-actin-R (5′- AGCACTGTGTTGGCGTACAG -3′) produced by MWG Eurofins (Ebersberg, Germany) and COX-2 (PTGS2) primers were purchased from Biomol (Hamburg, Germany). All samples were analysed in duplicates and the amounts of expressed mRNA were normalized to *β*-actin mRNA expression.

### siRNA treatment

For siRNA transfection, cells grown in 12-well plates were submitted to lipofection using 6 *μ*l of the HiperFect reagent (Qiagen, Hilden, Germany) and 150 ng/well of either negative control siRNA, RelA, RelB, c-Rel, COX-2 or NFATc2 siRNA. For each gene target at least two siRNA targeting different sequences were used: Rel-genes Set 1: RelA-HSS109159, RelB-HSS109162, c-Rel-HSS109157; Rel-genes Set 2: RelA-HSS109161; RelB-HSS184267, c-Rel-HSS109157; NFATc2 HSS107110, HSS107111 (all from Invitrogen, Darmstadt, Germany); and COX-2: s11472, s11474 (Ambion, Darmstadt, Germany).

### Genome-wide transcriptome profiling

Total RNA was isolated, processed and hybridized to an Affymetrix Human Gene 1.0 ST array according to the manufacturer's guidelines (Qiagen RNEasy and Affymetrix HG 1.0 ST). Data were normalized using RMA (R, Bioconductor). Fold changes were calculated based on the ratios of the medians. Transcripts were categorized as present when their median expression in at least one experimental group was above the 5th percentile of all exonic control signals integrated on the employed Affymetrix 1.0 ST array. Transcripts with fold changes >2.5 or <−2.5 were considered differentially expressed in this exemplary approach.

### Cluster analysis

Cluster analysis was performed using TIBCO Spotfire IBD (Tibco, Boston, MA, USA) using the unweighted pair group method with arithmetic mean as a cluster algorithm and using correlation as a distance measure. All transcripts were subjected to *z*-score normalization before cluster analysis. For the cluster analysis, only the top 50 characterized genes, ranked by fold change, were selected.

### Caspase-3/-7 assay

Apoptosis induced by Killer-TRAIL was determined by the measurement of caspase-3/-7 activity (Promega) according to the manufacturer's instructions and as described.^65^ All assays were carried out in duplicates. Caspase-3/-7 activity was normalized to the protein content of the analysed cell lysates.

### Sub-G1 apoptosis assay (Nicoletti)

For sub-G1 apoptosis assays cells were trysinized, washed with PBS and stained with propidium iodide solution (50 *μ*g/ml propidium iodide, 0.1% sodium citrate, 0.1% Trition X-100; all from Sigma, Munich, Germany). After an incubation for 48 h at 4 °C, flow cytometry was carried out on a FACSscan flow cytometer (BD, Heidelberg, Germany) and cells with high fragmented DNA content (sub-G1) were regarded as being apoptotic.

### ChIP assay

ChIP assays were performed by using SimpleChip Enzymatic Chromatin IP Kits from Cell Signalling (Boston, MA, USA) due to the manufacturer's instructions. DNA from Panc1 or Patu8988t cells was submitted to immunoprecipitation with 2 *μ*g of mouse IgG (Upstate, Darmstadt, Germany), anti-c-Rel (Santa Cruz Biotechnology), anti-Rbp1, anti-NFATc2 or rabbit IgG (all Cell Signalling). Primers for real-time PCR: NFATc2-F (5′-CGAACCAGGGTCTAGACAAG-′3); NFATc2-R (5′-CCATGCAGGTGTCTGAAGTA -′3) (from MWG Eurofins).

### Statistics

Data represent the mean±S.D. and were analysed by Student's *t*-test, *P*-values <0.05 were considered as statistically significant and indicated by an asterisk.

## Abbreviations

PDAC: pancreatic ductal adenocarcinoma
TRAIL: tumour necrosis factor-related apoptosis-inducing ligand
